# Prognostic value of GLUT-1 expression in oral squamous cell carcinoma: A prisma-compliant meta-analysis

**DOI:** 10.1097/MD.0000000000005324

**Published:** 2016-11-11

**Authors:** Chen-Xi Li, Jia-Lin Sun, Zhong-Cheng Gong, Zhao-Quan Lin, Hui Liu

**Affiliations:** a Department of Oral and Maxillofacial Oncology Surgery, Stomatological Medical Center, The First Affiliated Hospital of Xinjiang Medical University; b School of Public Health, Xinjiang Medical University; c Department of Oral and Maxillofacial Surgery, The First Affiliated Hospital of Xinjiang Medical University, Urumqi, Xinjiang Autonomous Region, China.

**Keywords:** biomarker, GLUT-1, meta-analysis, oral squamous cell carcinoma, prognosis

## Abstract

**Background:**

A variety of studies have evaluated the correlation between glucose transporter-1 (GLUT-1) expression and prognosis of oral squamous cell carcinoma (OSCC); however, the results were inconsistent and inconclusive. A meta-analysis was performed to assess the prognostic significance of GLUT-1 in OSCC.

**Methods:**

Electronic databases of PubMed, Embase, and Web of Science were searched for relevant studies. The last search was updated on July 2016. Odds ratio (OR) and 95% confidence interval (CI) were pooled to evaluate the relationship between GLUT-1 and clinical features and hazard ratio (HR) and 95% CI were combined to measure the effect of GLUT-1 on overall survival (OS). *P* value < 0.05 was considered as statistically significant.

**Results:**

A total of 13 studies with 1301 subjects were included for meta-analysis. The pooled data showed that high GLUT-1 expression was associated with advanced tumor stages (n = 7, OR = 2.99, 95% CI: 2.01–4.46, *P* < 0.001), higher tumor grade (n = 5, OR = 3.34, 95%CI: 1.12–9.94, *P* = 0.031), tumor size (n = 5, OR = 3.36, 95%CI: 2.04–5.51, *P* < 0.001), lymph node metastasis (n = 5, OR = 3.15, 95%CI: 1.89–5.25, *P* < 0.001), tobacco use (n = 3, OR = 2.18, 95%CI: 1.18–4.01, *P* = 0.013), and distant metastasis (n = 2, OR = 3.06, 95%CI: 1.19–7.9, *P* = 0.02). Furthermore, increased GLUT-1 expression was also correlated with shorter OS (n = 8, HR = 1.88, 95%CI: 1.51–2.33, *P* < 0.001). No significant publication bias was detected in this meta-analysis.

**Conclusion:**

GLUT-1 overexpression was in connection with aggressive clinical features and worse OS in OSCC. However, further studies are still needed to verify whether GLUT-1 could serve as a prognostic biomarker for OSCC.

## 1 Introduction

Oral cancer is a serious problem in many parts of the world with an estimated incidence of 275,000 annually.^[1]^ Oral squamous cell carcinoma (OSCC) accounts for 90% of all malignant oral lesions and occurs with a growing incidence rate in young and middle-age population.^[2]^ Despite treatment modalities including surgical resection, radiotherapy, and chemotherapy have improved a lot in the past several decades, the long-term survival of OSCC patients was still poor because of a high rate of locoregional recurrence and new malignant conversions.^[3,4]^ To more precisely identify high-risk patients and further to predict clinical outcomes, reliable and novel prognostic markers are imperatively needed.

As first described by Otto Warburg >90 years ago, 1 important characteristic of malignant cells is an increased glucose uptake and enhanced glycolytic metabolism of carbohydrates, even in the presence of oxygen.^[5]^ Consequently, a variety of malignant tumors showed overexpression of glucose transporters (GLUTs). The GLUTs family is composed of 13 members,^[6]^ among which, glucose transporter-1 (GLUT-1) is the first to be cloned and the dominant one. In most tissues, GLUT-1 was expressed at a very low level, whereas it was found to be overexpressed in various malignant tumors including nonsmall cell lung cancer,^[7]^ colorectal cancer,^[8]^ gastric cancer,^[9]^ breast cancer,^[10]^ and OSCC.^[11–13]^ In addition, many studies reported that GLUT-1 overexpression was an indicator of poor survival outcomes in OSCC and was also correlated with status of metastasis and other clinical parameters.^[11–14]^ For example, Kunkel et al^[11]^ reported that high GLUT-1 expression was a biomarker in both early (Stages I and II) OSCC (*P* = 0.045) and advanced (Stages III and IV) OSCC (*P* = 0.029). Meanwhile, Ayala and colleagues^[13]^ also identified GLUT-1 as an indicator for poor overall survival (*P* = 0.006) in their study. Swartz et al^[15]^ also found significantly prognostic role of GLUT-1 in OSCC by univariate overall survival analysis (*P* = 0.026). In contrast, other studies did not find any significant association between GLUT-1 and outcome of OSCC patients.^[16,17]^ For example, Eckert et al^[16]^ showed that increased GLUT-1 expression did not significantly correlate with poor survival in OSCC (*P* = 0.171). Similarly, Han and coworkers^[17]^ also failed to identify GLUT-1 as a marker for survival prediction in their study (*P* = 0.91). Because of limited sample size and other heterogeneity in these clinical studies, the conclusions should be further analyzed by quantitative method to form more reliable results. Therefore, in the current study, a meta-analysis was conducted to systematically and quantitatively synthesize the results from relevant studies to elucidate the relationship between GLUT-1 expression and clinical features as well as overall survival (OS) in OSCC. To our knowledge, this was the first meta-analysis reporting the prognostic role of GLUT-1 in OSCC to date.

## 2 Methods

### 2.1 Publication search

This meta-analysis was conducted according to the PRISMA guidelines.^[18]^ All relevant literatures were comprehensively searched through electronic databases of PubMed, Embase, and Web of Science and the last search was updated on July, 2016. The following search terms were used: “glucose transporter-1” and “oral squamous cell carcinoma.” Ethical approval was waived because this study was performed on basis of published data and did not have direct access to patient information.

### 2.2 Selection criteria

The inclusion criteria were: (i) the diagnosis of OSCC was pathologically confirmed; (ii) investigated the association between GLUT-1 and clinicopathological factors or OS; (iii) GLUT-1 expression was determined by immunohistochemistry (IHC); (iv) sufficient data were provided to allow comparison; (v) published in English as full-text papers. Studies meeting the following criteria were excluded: (i) reviews, meeting abstracts, letters or case reports; (ii) necessary information was lacking; (iii) nonhuman studies; (iv) if multiple studies were reported based on the same patient group, the most comprehensive one was included.

### 2.3 Data extraction

Two investigators independently extracted the following information from each eligible study: first author, year of publication, study country, patient number, gender, age, detection method, cut-off value, positive rate, clinical parameters, and survival outcomes. Any discrepancies between the 2 investigators were resolved by discussion.

### 2.4 Statistical analysis

The impact of GLUT-1 expression on clinical features were evaluated by pooling odds ratio (OR) and 95% confidence interval (CI) from included studies. Hazard ratio (HR) and 95% CI were combined to measure the effect of GLUT-1 on OS. If HRs and 95% CIs were not directly reported in text, these values were calculated from Kaplan–Meier survival curves according to method described by Parmar et al.^[19]^ Heterogeneity among included studies was assessed by Cochran's *Q* test^[20]^ and the Higgins *I*^2^ statistic.^[21]^ A *P*-value for heterogeneity (*P*_*h*_) >0.1 and *I*^2^ < 50% indicate nonsignificant heterogeneity and the fixed-effects model (Mantel Haenszel method) was used for analysis. Otherwise, the random-effects model (DerSimonian Laird method) was utilized. Potential publication bias was measured by both Begg's test and Egger's test. *P* < 0.05 was considered as statistically significant. All analyses were carried out using STATA version 12.0 (Stata Corporation, College Station, TX).

## 3 Results

### 3.1 Study selection and characteristics of included studies

**Figure 1:**
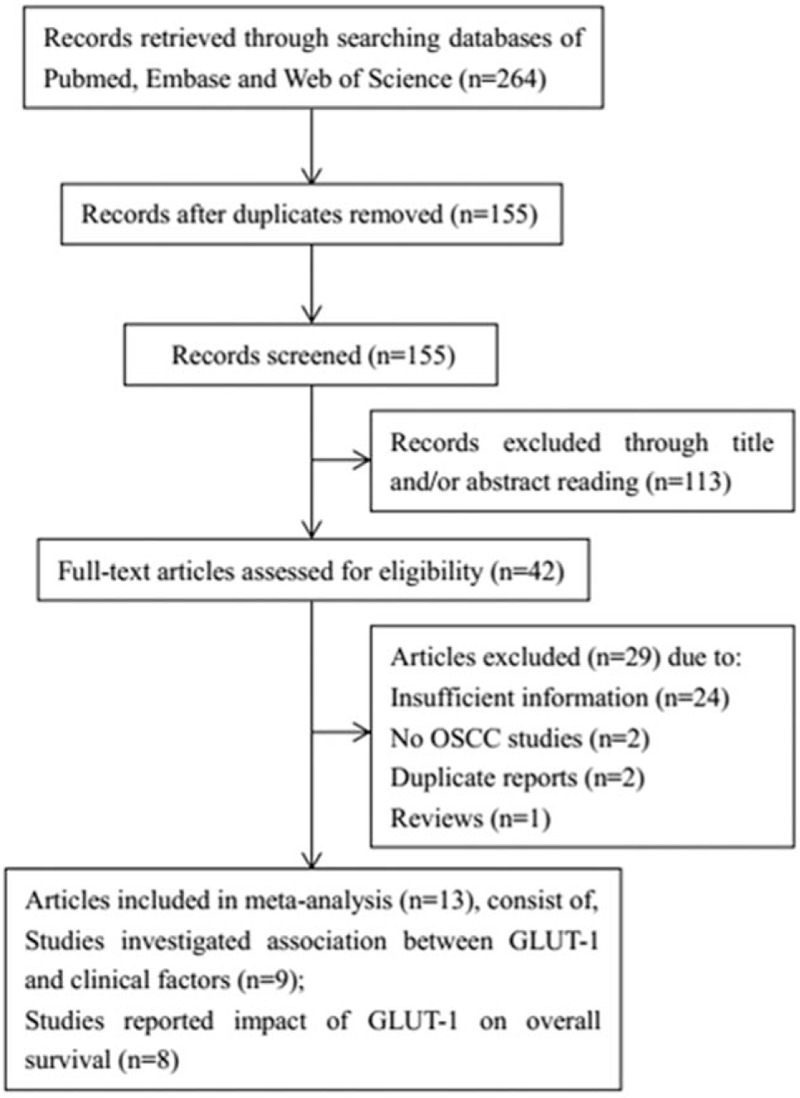
The flow graph of literature search and selection of articles.

A total of 264 studies were identified by initial searching and 155 were left after duplicate records were removed. By reading title and/or abstract, 113 records were excluded and the remaining 42 records were read by full-text. Then 29 records were excluded because they lacked necessary information, did not study on OSCC patients, and were duplicate reports on the same patients or reviews. Finally, 13 studies^[11–17,22–27]^ with 1301 subjects were included for meta-analysis. The flowchart of study selection was illustrated in Fig. 1. Nine studies^[12–14,16,23–27]^ investigated the relationship between GLUT-1 and clinical parameters in OSCC and 8 studies^[11–17,23]^ reported the association between GLUT-1 and OS. The included studies were published from 2003 to 2016 and the sample sizes ranged from 24 to 274. All studies used IHC to detect GLUT-1 expression. Detailed information of all included studies was summarized in Table 1.

**Table 1:**
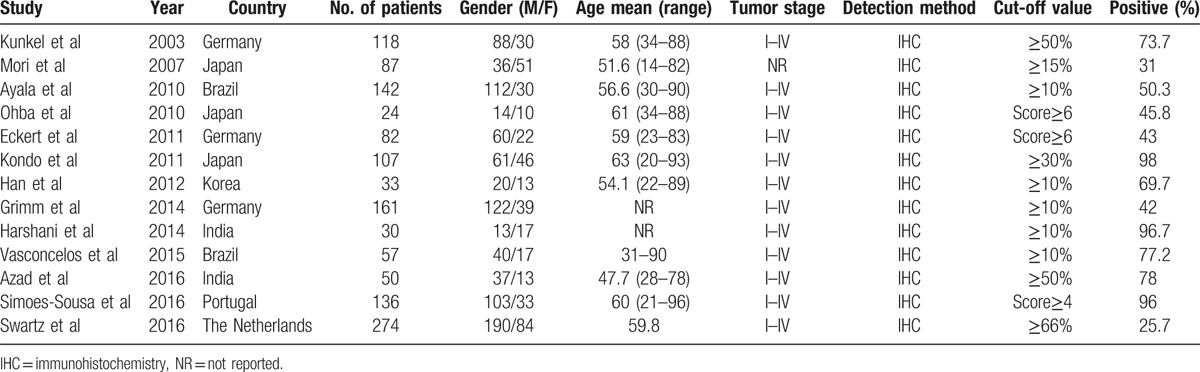
Basic information of included studies.

### 3.2 GLUT-1 and clinical characteristics in OSCC

**Figure 2:**
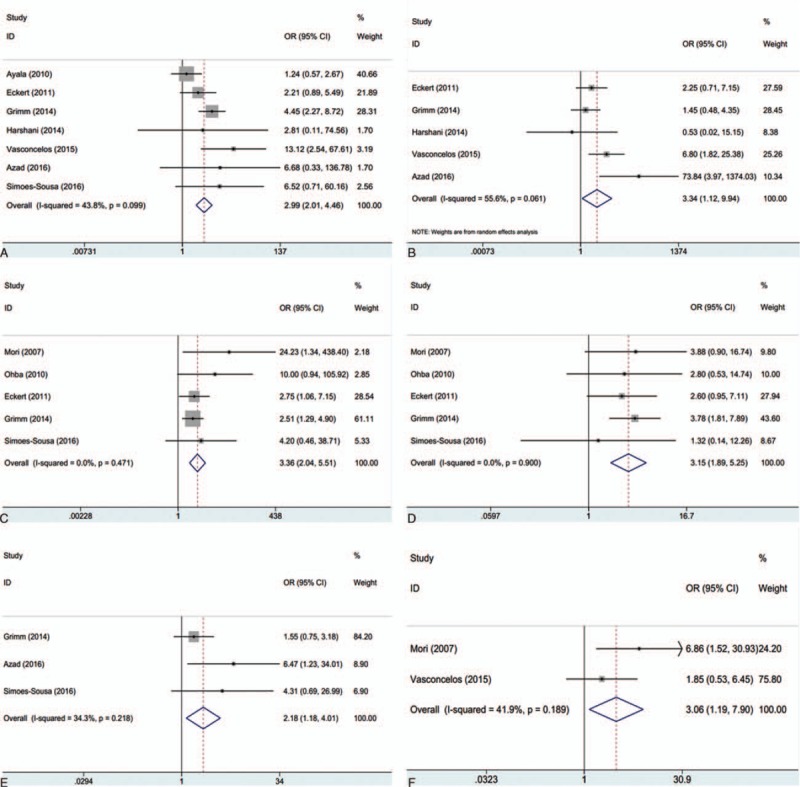
The association between GLUT-1 expression and (A) tumor stage (OR = 2.99, 95% CI: 2.01–4.46, *P* < 0.001), (B) tumor grade (OR = 3.34, 95%CI: 1.12–9.94, *P* = 0.031), (C) tumor size (OR = 3.36, 95%CI: 2.04–5.51, *P* < 0.001), (D) lymph node metastasis (OR = 3.15, 95%CI: 1.89–5.25, *P* < 0.001), (E) tobacco use (OR = 2.18, 95%CI: 1.18–4.01, *P* = 0.013), and (F) distant metastasis (OR = 3.06, 95%CI: 1.19–7.9, *P* = 0.02) in OSCC patients. CI = confidence interval, GLUT-1 = glucose transporter-1, OR = odds ratio, OSCC = oral squamous cell carcinoma.

The clinical parameters were: gender (male vs female), age (≥55 years vs < 55 years), tumor stage (III+IV vs I+II), tumor grade (G3+G4 vs G1+G2), tumor size (T3+T4 vs T1+T2), lymph node metastasis (yes vs no), alcohol use (yes vs no), tobacco use (yes vs no), local recurrence (yes vs no), and distant metastasis (yes vs no). The results pooled from 7 studies^[13,16,23–27]^ showed that patients with advanced stages (III+IV) had high GLUT-1 expression than early stages (I+II) patients: OR = 2.99, 95% CI: 2.01–4.46, *P* < 0.001 (Fig. 2A). Patients with higher tumor grade (G3+G4) had increased GLUT-1 expression than patients with lower tumor grade (G1+G2): OR = 3.34, 95%CI: 1.12–9.94, *P* = 0.031 (Fig. 2B). In addition, GLUT-1 overexpression was also associated with tumor size (n = 5, OR = 3.36, 95%CI: 2.04–5.51, *P* < 0.001; Fig. 2C), lymph node metastasis (n = 5, OR = 3.15, 95%CI: 1.89–5.25, *P* < 0.001; Fig. 2D), tobacco use (n = 3, OR = 2.18, 95%CI: 1.18–4.01, *P* = 0.013; Fig. 2E), and distant metastasis (n = 2, OR = 3.06, 95%CI: 1.19–7.9, *P* = 0.02; Fig. 2F). However, GLUT-1 was found to have nonsignificant correlation to gender (n = 6, OR = 0.97, 95%CI: 0.64–1.47, *P* = 0.884), age (n = 3, OR = 1.94, 95%CI: 0.41–9.25, *P* = 0.407), alcohol use (n = 3, OR = 1.37, 95%CI: 0.82–2.28, *P* = 0.224), or local recurrence (n = 3, OR = 2.07, 95%CI: 0.53–8.13, *P* = 0.297).

### 3.3 GLUT-1 and overall survival in OSCC

**Figure 3:**
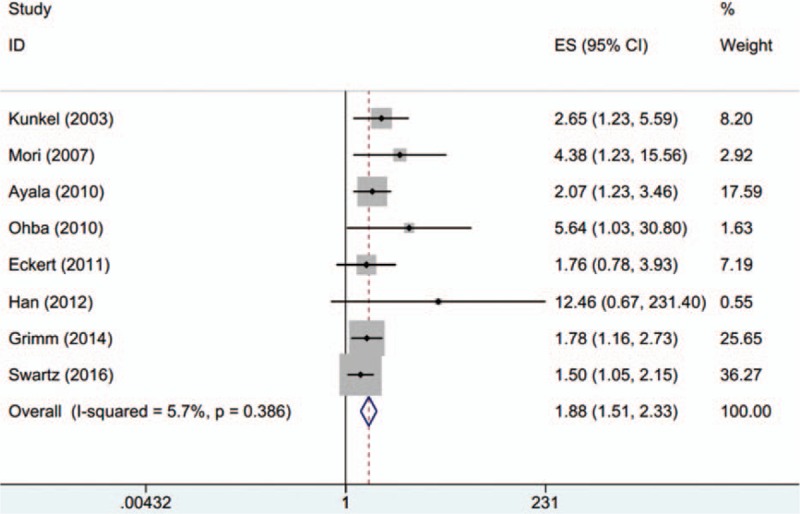
The correlation between GLUT-1 and overall survival in OSCC (HR = 1.88, 95%CI: 1.51–2.33, *P* < 0.001). GLUT-1 = glucose transporter-1, HR = hazard ratio, OSCC = oral squamous cell carcinoma.

Eight studies^[11–17,23]^ involving 921 patients exploited the prognostic role of GLUT-1 on OS. Because nonsignificant heterogeneity (*I*^2^ = 5.7%, *P*_*h*_ = 0.386) was detected, the fixed-effects model was used. The combined results were: HR = 1.88, 95%CI: 1.51–2.33, *P* < 0.001 (Fig. 3). The data indicated that elevated GLUT-1 was associated with shorter OS in OSCC patients.

### 3.4 Sensitivity analysis

**Figure 4:**
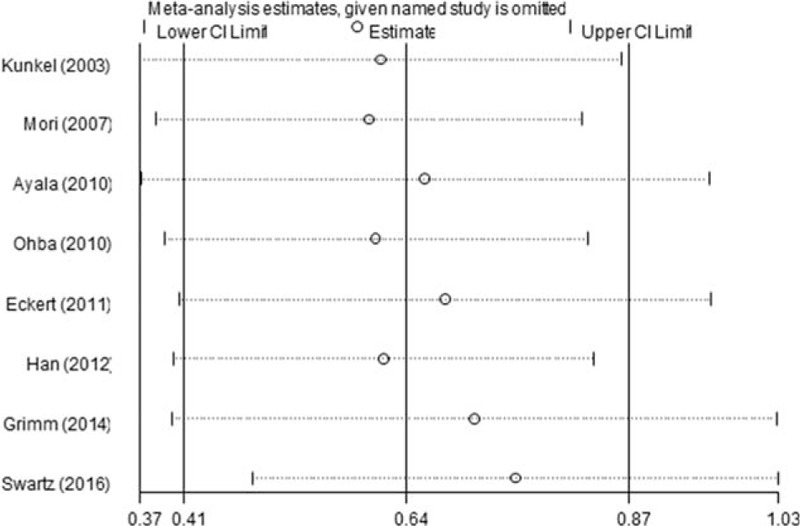
Sensitivity analysis of the HRs for overall survival. There was no substantial change by sequential omission of each study. HRs = hazard ratios.

Sensitivity analysis was performed by sequential omission of each single study to evaluate its impact on total results. As shown in Fig. 4, after excluding each individual study 1 by 1, the pooled results were not substantially changed, confirming the reliability of the data.

### 3.5 Publication bias

**Figure 5:**
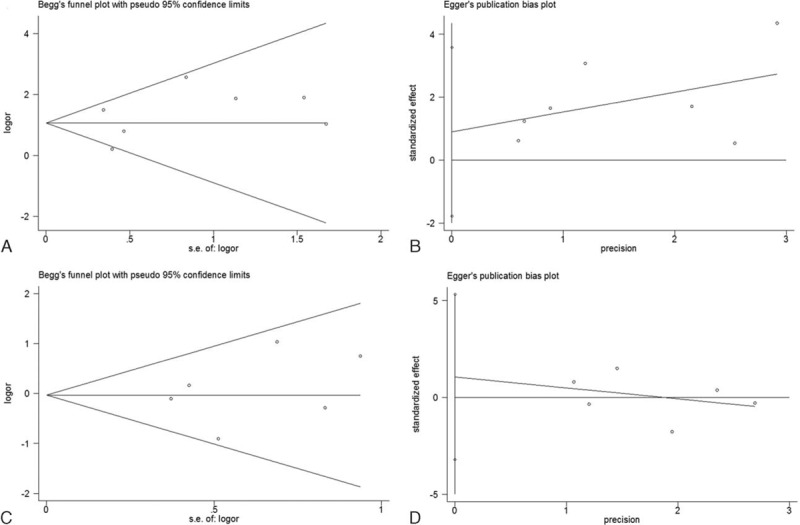
Publication bias tested by Begg's test and Egger's test. (A) Begg's test for tumor stage, (B) Egger's test for tumor stage, (C) Begg's test for tumor grade, (D) Egger's test for tumor grade.

Begg's test and Egger's test were simultaneously adopted to examine publication bias. The results revealed that there was no significant publication bias for all analyses (Table 2 and Fig. 5).

**Table 2:**
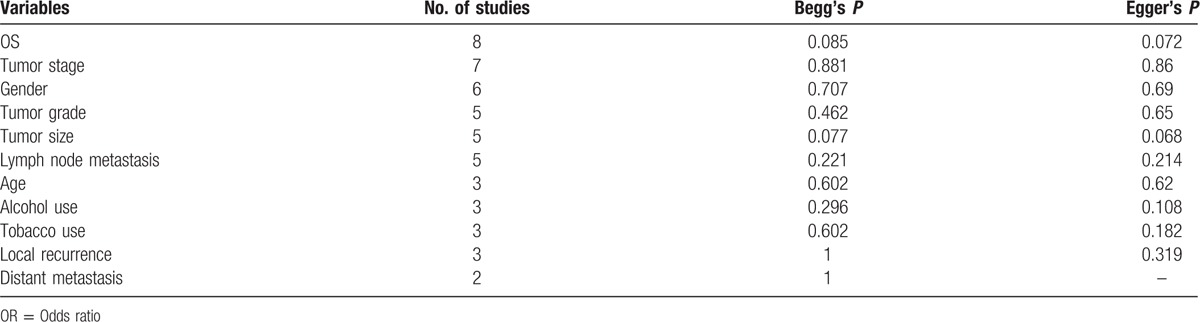
Publication bias tested by Begg's test and Egger's test in meta-analysis.

## 4 Discussion

OSCC is the most common form of oral cancer with a 5-year overall survival rate of about 40% to 50%.^[28]^ Traditional predictive factors such as TNM system and histopathological differentiation were inadequate to provide sufficient information on prognosis. In the present study, we used the analytic technique of meta-analysis to aggregate data from 13 studies. The pooled results demonstrated that GLUT-1 overexpression was positively with advanced tumor stage, higher tumor grade, larger tumor size, lymph node metastasis, tobacco use, and distant metastasis. Furthermore, the combined data also disclosed that increased GLUT-1 expression was an indicator for shortened OS in OSCC. To our knowledge, the present study was the first meta-analysis showing the prognostic significance of GLUT-1 in OSCC.

GLUT-1 was considered to provide the fundamental activity of glucose transport in glucose metabolism.^[29]^ GLUT-1 possesses high affinity and provides potential energy for cellular growth.^[6]^ Overexpression of GLUT-1 could facilitate growth and proliferation of tumor cells through supporting the high metabolic consumption in hypoxic tumor microenvironment.^[9]^ A number of studies reported the direct link between high GLUT-1 levels and malignant conversion process.^[11–14,16,17,22,30]^ The activity of GLUT-1 was regulated by oncogenes and growth factors.^[11,31]^ Changes of GLUT-1 levels can be influenced by growth rate, oxygen supply, and malignant transformation.^[32]^ In the current meta-analysis, we found that GLUT-1 overexpression was in association with parameters reflecting high aggressive and invasive potential of OSCC. When tumor cells invade to normal tissues and metastasize to distant locations, they are often lacked of energy; therefore, the GLUT-1 levels are consequently upregulated to support the metabolic consumption. This is the potential mechanism underlying the correlation between GLUT-1 and poor prognosis in OSCC.

The findings in this meta-analysis were also related to results from other studies concerning OSCC.^[33,34]^ A recent meta-analysis reported that hypoxia inducible factors (HIFs) were overexpressed in OSCC and were connected with increased risk of mortality.^[33]^ As HIFs are crucial markers which regulate cellular responses under hypoxic conditions. HIFs and GLUT-1 were found to be both overexpressed in OSCC.^[25]^ The relevance between HIFs and GLUT-1 could support the important role of energy supply for tumor cells. In the present meta-analysis, we included studies detecting GLUT-1 by unified method, IHC, to reduce heterogeneity. Furthermore, both sensitivity analysis and publication bias test were conducted and the results confirmed the robustness of our results.

There are some limitations need to be considered. First, the number of studies and sample size were still relatively small. Second, subgroup analysis was not performed for OS analysis because the heterogeneity was slight (*I*^2^ = 5.7%, *P*_*h*_ = 0.386), and the studies were homogeneous. Therefore, further subgroups could not be separated. Third, as papers with positive results were prone to be published, so negative findings have less chance to be obtained and included in this meta-analysis, which may cause overestimation of prognostic role of GLUT-1 in OSCC.

In summary, results from the current meta-analysis showed that GLUT-1 overexpression was in connection with aggressive clinical features and worse OS in OSCC. However, due to several limitations, further studies are still needed to verify whether GLUT-1 could serve as a prognostic biomarker for OSCC.
