# Prognostic value of GLUT-1 expression in oral squamous cell carcinoma: A prisma-compliant meta-analysis: Erratum

**DOI:** 10.1097/MD.0000000000006497

**Published:** 2017-03-24

In the article “Prognostic value of GLUT-1 expression in oral squamous cell carcinoma: A prisma-compliant meta-analysis”,^[1]^ which appeared in Volume 95, Issue 45 of *Medicine*, the affiliation for Drs. Gong and Lin was given incompletely. The full affiliation for these authors should read as follows: Department of Oral and Maxillofacial Oncology Surgery, Stomatological Medical Center, The First Affiliated Hospital of Xinjiang Medical University.
